# Association of *CYP1B1* Polymorphisms with Breast Cancer: A Case-Control Study in the Han Population in Ningxia Hui Autonomous Region, P. R. China

**DOI:** 10.4137/bmi.s4094

**Published:** 2010-02-12

**Authors:** Haiyan Jiao, Chunlian Liu, Weidong Guo, Liang Peng, Yintao Chen, Francis L. Martin

**Affiliations:** 1 Department of Medical Genetics and Cell Biology, Ningxia Medical University, Yinchuan, Ningxia, 750004, P. R. China; 2 Key Laboratory of Reproduction and Genetics of Ningxia Hui Antonomous Region, Yinchuan, Ningxia, 750004, P. R. China; 3 Department of Reproduction, Center of Gynaecology and Obstetrics, Affiliated Hospital of Ningxia Medical University, Yinchuan, Ningxia, 750004, P. R. China; 4 Department of Surgical Oncology, Affiliated Hospital of Ningxia Medical University, Yinchuan, Ningxia, 750004, P. R. China; 5 Centre for Biophotonics, Lancaster Environment Centre, Lancaster University, Bailrigg, Lancaster LA1 4YQ, UK. Email: f.martin@lancaster.ac.uk

**Keywords:** breast cancer, CYP1B1, Han population, Ningxia (China), phase I metabolism, polymorphism

## Abstract

Studies investigating possible associations between cytochrome P4501B1 (*CYP1B1*) polymorphisms and breast cancer risk have been inconsistent. We set out to ascertain whether there might be an association between polymorphisms in exon 2 (codon 119, G→T) and exon 3 (codon 432, G→C) of *CYP1B1* and breast cancer in a Chinese Han population in the rural region of Ningxia. Using an allele-specific polymerase chain reaction method and direct DNA sequencing, the presence or absence of the two *CYP1B1* polymorphisms was investigated. Genotype and allele frequencies were analyzed in breast cancer cases (n = 152) and healthy age-matched controls (n = 156). The odds ratio (OR) of 119G→T or 432G→C in breast cancer cases and controls was 3.3 (95% CI: 1.28 to 8.28) and 2.8 (95% CI: 1.04 to 7.51), respectively. In addition, the OR for people with both polymorphisms (119T and 432C) was 4.69 (95% CI: 1.97 to 11.19). Our results suggest that certain polymorphisms in the *CYP1B1* gene might increase risk for breast cancer among Han Chinese, perhaps because they influence the efficiency of *CYP1B1* bio-transformation of oestrogens or pro-carcinogens into DNA-reactive electrophiles that may act as cancer-initiating agents.

## Background

Breast cancer remains a major cause of morbidity and mortality worldwide with incidence rates ranging from 27/100,000 in Asian countries to 97/100,000 among US Causcasian women, but its aetiology remains unclear. Only a small proportion of cases (<10%) may be due to germline mutations in highly-penetrant susceptibility genes e.g. *BRCA1/2*.^1^ The majority of cases are sporadic and their causes are obscure, although cumulative hormonal exposure and environmental influences appear to be risk factors.^2^,^3^ Cytochrome P4501B1 (*CYP1B1*) catalyzes the formation of genotoxic 4-hydroxy oestradiol (4-*OH* E_2_), and bio-activates environmental pro-carcinogens [e.g. polycyclic aromatic hydrocarbons (PAHs), heterocyclic aromatic amines (HAAs)].^4^–^7^ The expression of *CYP1B1* and the formation of 4-*OH* E_2_ have been associated with oestrogen-related tumours in multiple tissues and species.^8^–^10^ The 4-*OH*E_2_ catechol metabolite has the highest carcinogenic as well as oestrogenic activity and is found in higher quantities in breast cancer tissue than other 17β-oestradiol (E_2_) metabolites.^11^

*CYP1B1* is genetically polymorphic, and sequence variations in the gene may be related to risk of breast cancer in some populations.^12^–^14^ The human *CYP1B1* gene, located at the 2p21–22 region, consists of three exons, one of which is noncoding, and two introns.^15^ Two common polymorphisms of *CYP1B1* in exon 2 (codon 119, Ala→Ser) and exon 3 (codon 432, Val→Leu) encode the heme-binding domain; variant alleles probably exhibit altered activity.^6^ Therefore, genetic polymorphisms at loci encoding *CYP1B1* may explain why individuals vary in their susceptibility to the carcinogenic effects of environmental chemicals.

Studies reporting the association between *CYP1B1* polymorphisms and breast cancer have involved different populations but, have given rise to often inconsistent and/or contradicting findings even within the same ethnic groups.^12^–^14^,^16^–^19^ We undertook a case-control study of a genetically-uniform population of Han Chinese in the Ningxia Hui Autonomous Region of P. R. China in which genotype and allele frequency associations between *CYP1B1* polymorphisms [in exon 2 (codon 119, G→T) and exon 3 (codon 432, G→C)] with breast cancer patients and healthy controls were determined. Our results point to an association between these two polymorphisms and breast cancer in this study population.

## Material and Methods

### Study participants

From May 2005 to June 2006, a total of 152 sporadic breast cancer patients and 156 healthy controls were recruited at Affiliated Hospital of Ningxia Medical University. All participants provided written informed consent before participating in the study. This research programme was approved by the local ethics approvals board at Ningxia Medical University.

The 152 breast cancer cases were verified by histopathology with a single pathologist. A cohort of 156 controls confirmed to be free of any cancer based on a health examination were randomly matched to the breast cancer arm of the study, with an age difference that was no greater than 5 years (y). The status for oestrogen receptor (ER) and progesterone receptor (PR) was also collected. The controls were not related in any way with the patients and they had no family history of breast cancer. All participants were long-term residents of Ningxia who had no prior history of cancer.

### DNA isolation and analysis of single nucleotide polymorphisms (SNPs)

Blood samples (3–5 ml) were collected in Vacutainer tubes containing ethylenediaminetetraacetic acid (EDTA). Genomic DNA was extracted using standard phenol/chloroform methods. The sequences of primers for polymerase chain reaction (PCR) at each locus were synthesized by SBS-Biology (Beijing) and are listed in Table 1.

### Polymerase chain reaction (PCR) amplification

Genotyping assays for the two SNPs for *CYP1B1* were conducted using allele-specific (AS) PCR methods in a Biometra T Gradient Thermocycler (Bio-Rad Laboratories). Each PCR mixture (25 μl) contained 10 ng DNA, 1 × PCR buffer with 1.5 mmol/L MgCl_2_, 0.16 mmol/L of each dNTP, 0.4 μmol/L of each primer, and 1 unit of Hotstart *Taq* DNA polymerase (SBS-Biology). The reaction mixture was initially denatured at 95 °C for 5 min, followed by 30 cycles at 94 °C for 30 sec, 60 °C for 30 sec, and 72 °C for 45 sec. The PCR was completed by a final extension cycle at 72 °C for 5 min. The DNA fragments were then separated and visualized by electrophoresis on 2% agarose gels containing ethidium bromide.

### Statistical analysis

We determined whether *CYP1B1* genotype frequencies were in Hardy-Weinberg equilibrium using standard χ^2^-statistics. Odds ratios (ORs) and 95% confidence intervals (CIs) were calculated using SPSS (Version 11.5; Chicago, IL., USA). ORs were used to measure the strength of the association between *CYP1B1* genotypes and breast cancer risk. The χ^2^ test was used to compare the distributions of *CYP1B1* alleles and genotypes in cases and controls.

## Results

The breast cancer patients had an age range between 28 y and 75 y, with a mean (M) and standard deviation (SD) of 52.27 (10.28) y. The control cohort had an age range between 30 y and 70 y, with a M (SD) of 52.69 (11.25) y. There was no significant difference in the age distribution between the patient and control arms of the study.

**Figure 1.**
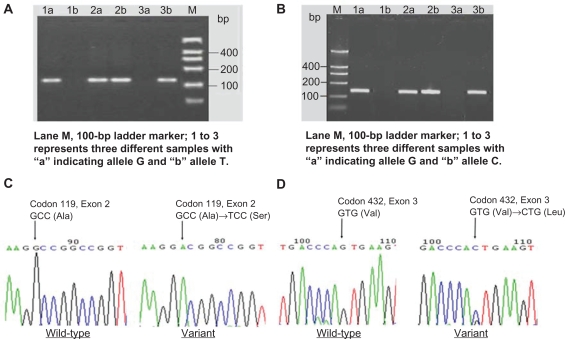
**A, B**) Representative samples for the genotyping for codons 119 and 432 of *CYP1B1*; **C**) Reverse sequence analysis to reveal a heterozygous G→T transition to result in substitution of Ala at codon 119; and, **D**) Direct sequence analysis to reveal a heterozygous G→C transition to result in substitution of Val at codon 432.

Representative samples for genotyping codons 119 and 432 of *CYP1B1* are shown in Figures 1A and B, and typical sequencing results are shown in Figures 1C and D. In tests to determine the genotypes of codon 119 in *CYP1B1*, the appearance of only the “a” band was identified with GG based on DNA fragment size, the appearance of both “a” and “b” bands was identified with GT, and the appearance of only a “b” band was identified with TT (Fig. 1A). For genotypes of codon 432 of *CYP1B1*, the appearance of only the “a” band corresponded to GG based on DNA fragment size, the appearance of both “a” and “b” bands corresponded to GC, and the appearance of only the “b” band corresponded to CC (Fig. 1B). Reverse sequence analysis of DNA revealed a heterozygous G→T transition at nucleotide 335 of *CYP1B1* (GenBank accession No. rs1056827), which results in a substitution of alanine (Ala) at codon 119 by a serine (Ser) (Fig. 1C). Direct sequence analysis of DNA also revealed a heterozygous G→C transition at nucleotide 1294 of *CYP1B1* (GenBank accession No. rs1056836), which causes a substitution of the valine (Val) at codon 432 by a leucine (Leu) (Fig. 1D).

We compared the distribution frequencies of the two SNPs for *CYP1B1* in breast cancer patients and the controls (Table 2). The frequencies of the G and T alleles on codon 119T/G of *CYP1B1* differed significantly between the two cohorts (*P* = 0.000) with an OR of 2.20 (95% CI: 1.49 to 3.26). The frequencies of G and C alleles on codon 432C/G of *CYP1B1* also showed significant differences between the two cohorts (*P* = 0.003), with an OR of 1.97 (95% CI: 1.25 to 3.11). The genotype frequencies at codons 119 and 432 for all subjects in this study are shown in Table 3. The distributions of the two polymorphisms were consistent with Hardy-Weinberg equilibrium (*P* > 0.05). There were significant differences in the GG, GT, and TT genotypes between the two cohorts (*P* = 0.001). The risk of breast cancer in individuals with genotypes of the homozygous TT mutation and heterozygous GT mutation was increased 3.26- and 2.32-fold, respectively, compared to the risk in individuals with the wild-type GG. There were significant differences in frequencies of the genotype GG, GC, CC between the two cohorts (*P* = 0.038) (Table 3). The risk of breast cancer in individuals with the homozygous CC mutation and the heterozygous GC mutation was increased 2.79- and 1.69-fold, respectively, compared to the risk in individuals with the wild-type GG.

We analyzed the effect of the combined 119/432 genotypes on breast cancer risk in the Ningxia Han population (Table 4). In our study, we observed individuals with the genotype of 119G/G-432C/G, 119T/T-432C/G, 119G/T-432C/C, or 119G/T-432G/G. Therefore it would seem that *CYP1B1* codons 119 and 432 are not in linkage disequilibrium. The OR for individuals possessing both 119T and 432C was nearly five times the OR for those possessing 119G/G-432G/G. No associations were found between these two variations and with ER or PR status.^20^

## Discussion

Individual susceptibility to cancer is likely to be influenced by the genotype for enzymes involved in the activation or detoxification of carcinogens. Polymorphic alleles of key candidate genes such as *CYP1B1*, which are involved in oestrogen and xenobiotic metabolism, may contribute to the different risks of breast cancer observed for different populations.^12^–^14^,^16^–^19^ In the present study, we investigated *CYP1B1* polymorphisms in a Ningxia Han Chinese population, and observed that the genotypes *119T/T* and *G/T* are present at a significantly higher frequency (*P* = 0.001) in breast cancer patients than in control participants. A difference between breast cancer patients and healthy participants was also found in the allelic distributions of codon 432. The combination of the two polymorphisms of *CYP1B1* investigated [in exon 2 (codon 119, G→T) and exon 3 (codon 432, G→C)] further increases the risk of breast cancer in this Ningxia Han (China) ethnic group.

Previous studies on the association between *CYP1B1* codon 119(G→T) and codon 432(G→C) and breast cancer in different populations have often given rise to conflicting or contradicting results. Our results on *CYP1B1* codon 119(G→T) confirm those of a study in a Japanese population.^21^ Nevertheless, we measured higher frequencies of the *CYP1B1* codon 119(G→T) “T” allele among controls (0.16) and cases (0.29) in the Ningxia Han than were reported among controls (0.12) and cases (0.16) in the Japanese study.^21^ Therefore, *CYP1B1* codon 119(G→T), which is thought to participate in the substrate recognition site 1 of *CYP1B1* protein, is significantly associated with breast cancer in both the Ningxia Han (China) population and the Japanese. This contradicts the protective effect reported for this polymorphism in studies carried out among Chinese women in Shanghai, Caucasian and African in U.S. (Nashville, Tennessee) and Polish women.^22^–^24^

The valine substitution is reported to be present at an allele frequency of 0.43 in Caucasians, 0.75 in African-Americans and 0.23 in Asians.^12^,^25^ No association between the *CYP1B1* Val432Leu polymorphism and breast cancer was observed in Asians (for *Val*/*Val* and *Val*/*Leu* combined, OR = 1.0, 95% CI: 0.8, 1.2). An inverse association was observed in a population of mixed/African origin (OR = 0.8, 95% CI: 0.7, 0.9). The pooled analysis suggested a possible association in Caucasians (for *Val*/*Val* and *Val*/*Leu* combined, OR = 1.5, 95% CI: 1.1, 2.1), with effect modification across age categories.^26^ Our finding of an association between *CYP1B1* codon 432 (G→C) and breast cancer is consistent with the results of several studies in Caucasians.^24^,^26^ In our study, the risk of breast cancer in individuals with the homozygous CC mutation or the heterozygous GC mutation was increased 2.79 (95% CI: 1.04 to 7.51)- and 1.69 (95% CI: 0.92 to 3.08)-fold, respectively, compared to that of individuals with wild-type GG. In one study of Caucasians, Listgarten et al^26^ found that the homozygous Leu/Leu mutation conferred a 3.30-fold higher risk of breast cancer (95% CI: 1.76 to 6.19), while the heterozygous *Val/Leu* genotype was associated with a 2.15-fold higher risk (95% CI: 1.31–3.52). In a study of a Shanghai population, Zheng et al^18^ found that women with the *Leu/Leu* genotype had a 2.3-fold [95% CI, 1.2–4.5] higher risk of breast cancer. Our study in the Ningxia Han population confirms these results, although we measured lower frequencies of the *CYP1B1* codon 432(G→C) “C” allele among controls (0.11) and cases (0.19) compared to those reported among controls (0.46) and cases (0.53) in the Shanghai Han population, respectively. Some studies in Caucasian populations and Asian populations^19^,^20^,^22^,^27^,^28^ have found no significant association between the *CYP1B1 Val/Leu* genotype and breast cancer risk. However, others have found a significant increase in the breast cancer risk for smoking women in a Finnish population (OR = 2.6, 95% CI 1.07–6.46), especially for smokers homozygous for the Leu allele (OR = 5.1, 95% CI 1.30–19.89, *P* for trend = 0.005).^13^

## Conclusion

Certain genotypes may contribute to variation in risk for breast cancer observed in different populations or ethnic groups. To evaluate this in a rural Ningxia Han population of P. R. China, we analysed the association between *CYP1B1* codon 119 (Ala→Ser) and codon 432 (Val→Leu) and risk of breast cancer. Our results show that the risk of breast cancer increases significantly (OR = 4.69, 95% CI 1.97–11.9) proportional to the number of variant alleles individuals possess. Han women living in the Ningxia province are relatively homogeneous in ethnic background, therefore eliminating some confounding effects. On the other hand, our study investigates a relatively small sample size in a case-control setting, which may make our risk estimates unstable. Nevertheless, our results are consistent with recent findings from *in vitro* and animal experiments implicating a potentially important role of *CYP1B1* in the aetiology of human breast cancer.^28^,^29^

Other factors such as the expressed profile of ER splice variants may play a hitherto under-recognised role in the modulation of breast cancer risk.^30^ However, we conclude that the functional polymorphism of *CYP1B1* may be a risk factor for breast cancer in the Ningxia Han population of China, and in at least some other populations. Further investigations involving larger sample sizes and other ethnic populations are needed in order to establish more completely the relationship between these *CYP1B1* SNPs and breast cancer risk.

## Figures and Tables

**Table 1.** The primer sequences in codons 119 and 432 of *CYP1B1*.

**Codon**	**Allele**	**Amino acid**	**Primers**	**Sequences**
119	GCC	Ala	*CYP1B1*-119G-F	GGCCTTCGCCGACCGGCCGG
	TCC	Ser	*CYP1B1*-119T-F	GGCCTTCGCCGACCGGCCGT
			*CYP1B1*-119-R	GAAGTTGCGCATCATGCTGT
432			*CYP1B1*-1294-F	ATGCGCTTCTCCAGCTTTGT
	CTG	Leu	*CYP1B1*-1294G-R	TCCGGGTTAGGCCACTTCAC
	GTG	Val	*CYP1B1*-1294C-R	TCCGGGTTAGGCCACTTCAG

**Abbreviations:** F, forward primer; R, reverse primer.

**Table 2.** Association of codons 119 and 432 of *CYP1B1* allele with breast cancer risk (Han population, Ningxia).

**Gene**	**Allele**	**No. of case (%) (n = 152)**	**No. of control (%) (n = 156)**	**χ^2^*P* value**	**OR (95% CI)**
A119S	G	217 (71.0)	263 (84.0)	15.5	
	T	87 (29.0)	49 (16.0)	0.000	2.20 (1.49 to 3.26)
V432L	G	245 (80.6)	278 (89.1)	8.78	
	C	59 (19.4)	34 (10.9)	0.003	1.97 (1.25 to 3.11)

**Table 3.** Association of codons 119 and 432 of *CYP1B1* genotype with breast cancer risk (Han population, Ningxia).

**Gene**	**Genotype**	**No. of case (%) (n = 152)**	**No. of control (%) (n = 156)**	**χ^2^*P* value**	**OR (95% CI)**
A119S	G/G	80 (52.6)	114 (73.1)		
	G/T	57 (37.5)	35 (22.4)	14.71	2.32 (1.40 to 3.86)
	T/T	15 (9.9)	7 (4.5)	0.001	3.26 (1.28 to 8.28)
V432L	G/G	107 (70.4)	128 (82.1)		
	G/C	31 (20.4)	22 (14.1)	6.55	1.69 (0.92 to 3.08)
	C/C	14 (9.2)	6 (3.8)	0.038	2.79 (1.04 to 7.51)

**Table 4.** Association between combined *CYP1B1* 119–432 genotypes and breast cancer (Han population, Ningxia).

**Combined genotype**	**No. of case (%) (n = 152)**	**No. of control (%) (n = 156)**	**OR (95%)**	***P* value**
G/G-G/G	57 (37.5)	93 (59.6)	–	–
G/G-C/G	19 (12.5)	16 (10.3)	1.94 (0.92 to 4.07)	0.078
G/T-G/G	41 (27.0)	27 (17.3)	2.48 (1.38 to 4.46)	0.002
G/T-C/G	11 (7.2)	5 (3.2)	3.59 (1.19 to 10.86)	0.017
Any T-G/G	52 (34.2)	32 (20.5)	2.65 (1.53 to 4.60)	0.000
Any T-C/G	15 (9.9)	6 (3.8)	4.08 (1.50 to 11.12)	0.004
G/G-any C	25 (16.5)	20 (12.8)	2.04 (1.04 to 4.00)	0.036
G/T-any C	16 (10.5)	7 (4.5)	3.73 (1.45 to 9.62)	0.004
Any T-any C	23 (15.1)	8 (5.1)	4.69 (1.97 to 11.19)	0.000

## Abbreviations

Ala: alanine;
AS-PCR: allele-specific polymerase chain reaction;
CI: confidence interval;
*CYP1B1*: cytochrome P4501B1;
E_2_: 17β-oestradiol;
EDTA: ethylenediaminetetraacetic acid;
ER: oestrogen receptor;
HAA: heterocyclic aromatic amine;
4-*OH* E_2_: 4-hydroxy oestradiol;
Leu: leucine;
M: mean;
OR: odds ratio;
PAH: polycyclic aromatic hydrocarbon;
PCR: polymerase chain reaction;
PR: progesterone receptor;
Ser: serine;
SD: standard deviation;
SNP: single nucleotide polymorphism;
Val: valine;
y: years.
